# Several steps/day indicators predict changes in anthropometric outcomes: HUB City Steps

**DOI:** 10.1186/1471-2458-12-983

**Published:** 2012-11-15

**Authors:** Jessica L Thomson, Alicia S Landry, Jamie M Zoellner, Catrine Tudor-Locke, Michael Webster, Carol Connell, Kathy Yadrick

**Affiliations:** 1 USDA Agricultural Research Service, 284 Knapp Hall, Human Nutrition and Food, Louisiana State University, Baton Rouge, LA, 70803, USA; 2 Department of Nutrition and Food Systems, University of Southern Mississippi, 118 College Drive #5172, Hattiesburg, MS, 39406, USA; 3 Department of Human Nutrition, Foods, and Exercise, Virginia Tech, 1981 Kraft Drive, Blacksburg, VA, 24061, USA; 4 Walking Behavior Laboratory, Pennington Biomedical Research Center, 6400 Perkins Road, Baton Rouge, LA, 70808, USA; 5 School of Human Performance and Recreation, University of Southern Mississippi, 118 College Drive #5142, Hattiesburg, MS, 39406, USA

**Keywords:** Pedometer, Steps/day, Anthropometric measures, African American

## Abstract

**Background:**

Walking for exercise remains the most frequently reported leisure-time activity, likely because it is simple, inexpensive, and easily incorporated into most people’s lifestyle. Pedometers are simple, convenient, and economical tools that can be used to quantify step-determined physical activity. Few studies have attempted to define the direct relationship between dynamic changes in pedometer-determined steps/day and changes in anthropometric and clinical outcomes. Hence, the objective of this secondary analysis was to evaluate the utility of several descriptive indicators of pedometer-determined steps/day for predicting changes in anthropometric and clinical outcomes using data from a community-based walking intervention, HUB City Steps, conducted in a southern, African American population. A secondary aim was to evaluate whether treating steps/day data for implausible values affected the ability of these data to predict intervention-induced changes in clinical and anthropometric outcomes.

**Methods:**

The data used in this secondary analysis were collected in 2010 from 269 participants in a six-month walking intervention targeting a reduction in blood pressure. Throughout the intervention, participants submitted weekly steps/day diaries based on pedometer self-monitoring. Changes (six-month minus baseline) in anthropometric (body mass index, waist circumference, percent body fat [%BF], fat mass) and clinical (blood pressure, lipids, glucose) outcomes were evaluated. Associations between steps/day indicators and changes in anthropometric and clinical outcomes were assessed using bivariate tests and multivariable linear regression analysis which controlled for demographic and baseline covariates.

**Results:**

Significant negative bivariate associations were observed between steps/day indicators and the majority of anthropometric and clinical outcome changes (r = -0.3 to -0.2: P < 0.05). After controlling for covariates in the regression analysis, only the relationships between steps/day indicators and changes in anthropometric (not clinical) outcomes remained significant. For example, a 1,000 steps/day increase in intervention mean steps/day resulted in a 0.1% decrease in %BF. Results for the three pedometer datasets (full, truncated, and excluded) were similar and yielded few meaningful differences in interpretation of the findings.

**Conclusions:**

Several descriptive indicators of steps/day may be useful for predicting anthropometric outcome changes. Further, manipulating steps/day data to address implausible values has little overall effect on the ability to predict these anthropometric changes.

## Background

Walking for exercise remains the most frequently reported leisure-time activity [1], likely because it is simple, inexpensive, and easily incorporated into most people’s lifestyle. The multiple health benefits of walking are well established and include weight management [2], reduced blood pressure [2-4], and improved lipid profiles [5] and glucose tolerance/insensitivity [6-8]. Despite these well known benefits, 36% of adults in the United States (US) were classified as sedentary (defined as the accumulation of < 5,000 steps/day) in 2005–2006 [9]. The situation is even worse for African American adults since overweight and obesity rates exceed those of the general population [10], and they are much more likely to take fewer steps/day and therefore be classified as sedentary as compared to white adults [9].

Compared to accelerometers, pedometers are simple, convenient, and inexpensive tools that can be used to quantify step-determined physical activity. A relative lack of steps/day is used to infer a physically inactive lifestyle. Pedometers have been used to describe steps/day taken by various populations [11-15], explore steps/day values required to achieve health benefits [16], evaluate intervention effectiveness [17-19], and promote healthy lifestyles [20-24]. However, few studies have attempted to define the direct relationship between dynamic changes in pedometer-determined steps/day and changes in anthropometric and clinical outcomes. The direct connection between changes in steps/day and changes in targeted health outcomes is lacking for all races/ethnicities, including African Americans. Further, based on a review of 27 pedometer-based, physical activity interventions, the difference between steps/day values collected (for three to seven days) at baseline and post-intervention was used as a measure of change [25]. However, it is not clear that this is the ‘best’ indicator for predicting health outcome changes. Hence, the primary objective of this secondary analysis was to evaluate the utility of several descriptive indicators of pedometer-determined steps/day for predicting changes in anthropometric and clinical outcomes using data from a community-based walking intervention, HUB City Steps, conducted in a southern African American population. A secondary objective was to evaluate whether treating steps/day data for implausible values affected the ability of these data to predict intervention-induced changes in anthropometric and clinical outcomes.

## Methods

### Study design

The procedures followed in the HUB City Steps study were approved by the Institutional Review Board of the University of Southern Mississippi, Hattiesburg, Mississippi. Informed consent was obtained from all study participants. Although the study was designed in two phases, an initial six months of intervention followed by 12 months of maintenance, only the intervention phase is relevant to these analyses. The quasi-experimental intervention phase was designed to assess the effectiveness of intervention treatment on blood pressure (BP) as well as a variety of other clinical (e.g. lipids, glucose) and anthropometric (e.g. body mass index [BMI], waist circumference, percent body fat [%BF], fat mass, lean body mass) outcomes. Recruitment of study participants involved a variety of methods including flyers, word of mouth, and community events. Eligibility criteria included age (18 years or older), English speaker, non-institutionalized, and resident in Hattiesburg area. Individuals with BP ≥ 180/110 were disqualified from participation and directed to seek immediate medical attention. All other individuals were eligible for participation regardless of BP status and medication regimen. Briefly, the physical activity component of the intervention included walking coaches who provided support to encourage walking, goal setting, and submission of pedometer diaries; and monthly education sessions with group physical activity and sharing of successes and challenges. A more detailed description of the HUB City Steps methodology can be found elsewhere [26]. This secondary analysis is focused on data collected at baseline (January-February 2010) and at the six-month visit (July 2010) following the initial intervention phase.

### Measures

Questionnaire data included demographic characteristics, medical diagnoses, medications, and smoking. Anthropometric measures included height, weight, waist circumference, %BF, fat mass, and lean mass. Height was measured using a portable stadiometer and a Tanita scale (model TBF-310T, standard adult mode) was used to measure weight and %BF (based on bioelectrical impedance analysis) [27], and to calculate BMI. Clinical measures included systolic and diastolic blood pressure (SBP and DBP), lipids, and glucose. Systolic and diastolic blood pressures were assessed using an OMRON HEM-907XL automatic inflation sphygmomanometer, which has been used in large scale clinical trials [28,29]. Non-fasting cholesterol, triglycerides, and glucose were assessed using the Cholestech LDX Lipid Analyzer, which is a reliable alternative to the conventional biochemical routine [30]. As a measure of fitness, the self-paced 6-minute walk test (6MWT) was performed. The 6MWT is reliable and can discriminate between fitness levels in a high-functioning population [31]. A more detailed description of the HUB City Steps procedures for data collection can be found elsewhere [26].

Each participant was given a Yamax pedometer (model SW-701, Yamax Corporation, Tokyo, Japan) with instructions to wear the pedometer continuously during the entire intervention period on their waist during waking hours; to remove only upon showering, bathing or swimming; and to reset the pedometer to zero each morning. Participants had the option of recording their steps/day using weekly pedometer diary postcards or the intervention’s website. In either case, participant-recorded steps/day were uniformly added to the database. Participants’ steps/day were not assessed prior to the initiation of the study’s six-month intervention phase.

### Statistical analyses

All statistical analyses were performed using SAS® software, version 9.2 (SAS Institute Inc., Cary, NC). Three pedometer datasets, full, truncated, and excluded, were used to compute and compare associations between indicators of pedometer-determined steps/day and changes in anthropometric and clinical parameters. The steps/day plausibility range was defined as values falling between 500 and 30,000 steps/day inclusive [32]. Thus, the full dataset consisted of all values, regardless of their plausibility. In the truncated dataset, values falling above or below the plausibility range were truncated to the respective end points (i.e. steps/day < 500 recoded as 500; steps/day > 30,000 recoded as 30,000). In the excluded dataset, values falling above or below the plausibility range were excluded from analysis. All participants who reported steps/day for at least one intervention day were included in the analyses.

Several methodological approaches to computing descriptive indicators of steps/day were explored and included measures of central tendency, proportions, and categorical variables. Additionally, these indicators were computed over the entire intervention period (referred to as intervention indicators) and for differences between steps/day reported during the first two weeks of the intervention and the remaining weeks (3–27; referred to as change in intervention indicators). Positive changes represent an increase in steps/day between the initial and remaining weeks for the intervention period. Since steps/day were not collected prior to the intervention period, change in intervention steps/day indicators represent persistence in or maintenance of step-defined physical activity rather than a true change from a pre-intervention baseline. Intervention indicators included mean, median, proportion of reported days meeting 7,500 and 10,000 steps/day, and four- and six-category step-defined physical activity classifications (based on intervention mean steps/day). The six-category classification consisted of: basal (< 2,500 steps/day), limited (2,500 – 4,999), low active (5,000 – 7,499), somewhat active (7,500 – 9,999), active (10,000 – 12,499), and highly active (≥ 12,500) [33]. The four-category classification collapsed the basal and limited categories into a sedentary (< 5,000 steps/day) category, while the active and highly active categories were collapsed into a single active (≥ 10,000) category. Intervention change indicators included change in mean, median, and the four- and six-category step-defined physical activity classifications.

Descriptive statistics including means, standard deviations, frequencies, and percentages were used to summarize demographic characteristics, descriptive indicators of steps/day, and anthropometric and clinical outcomes. Chi square or Fisher’s exact tests (categorical variables) and two sample t tests (continuous variables) were used to test for significant differences between participant subgroups, study completers and non-completers. Study completers were defined as participants who provided both baseline and six-month follow-up measures, while study non-completers were defined as participants who provided only baseline measures. Pearson’s correlation coefficients were used to characterize bivariate relationships between steps/day indicators and changes in outcome variables. Multivariate linear regression models were built to determine if significant associations between the steps/day indicators and changes in outcome variables remained significant after controlling for covariates. The outcome (dependent) variables included changes in waist circumference, BMI, %BF, fat mass, total cholesterol, triglycerides, and glucose. The predictor (independent) steps/day variables included intervention and change in intervention mean and median steps/day, the proportion of reported days which met at least 7,500 or 10,000 steps/day, and changes in intervention four- and six-category step-defined physical activity classifications. Covariates included in the models were age, sex, marital status, education, income, smoking status, BMI, diagnosis of respective chronic condition (high cholesterol, high glucose), fitness (based on 6MWT), compliance with recording steps/day (based upon proportion of completed daily step logs), and baseline value of the respective outcome variable. For comparison and modeling purposes, marital status was categorized as married or not married (including widowed, divorced, separated, and never married); education was categorized as less than high school, high school graduate/GED, or some college (including trade or vocational school and any college regardless of degree); while income was treated as a continuous variable due to the relatively large number (n=12) of original categories. Because our preliminary analyses indicated that there were no confounding effects for medication change (only eight participants reported a change during the study), it was not included as a covariate in these analyses. Only data from study completers were included in the analyses involving associations with steps/day indicators. The significance level of the tests was set at 0.05 although trending (0.05 < P ≤ 0.10) associations were also reported.

## Results

### Participant characteristics

Of the 345 individuals from the Hattiesburg, Mississippi community who expressed interest and were screened for the study, 269 (78%) completed the baseline assessment, all of which were eligible, and were enrolled in HUB City Steps. At six-month follow-up, 190 (71%) participants were re-assessed. Baseline demographic and clinical characteristics are presented in Table 1 for the 269 participants. The majority of participants were African American (94%) and female (85%) with a mean age of 44 years. Less than half (42%) of participants were married, over three-fourths (79%) had some college education, while approximately one-fourth (26%) reported a household income greater than $50,000 per year. Very few (9%) participants currently smoked and diagnoses of chronic conditions ranged from 16% for high blood glucose to 42% for high blood pressure. Mean BMI was 35 kg/m^2^ and mean SBP and DBP were 126 and 83 mm Hg, respectively. Demographic and clinical comparisons between study completers and non-completers revealed no significant differences between the two groups for sex, marital status, education, smoking status, prevalence of self-reported chronic conditions (high blood pressure, high blood glucose, and high cholesterol), waist circumference, %BF, fat mass, lean body mass, blood pressures, total cholesterol, high density lipoprotein (HDL), low density lipoprotein (LDL), glucose, or 6MWT (data not shown). However, study completers were significantly older (46 vs. 40 years, respectively; p < 0.0001), had lower mean BMI (34 vs. 37 kg/m^2^, respectively; p = 0.0150), and had higher mean triglycerides (137 vs. 117 mg/dL, respectively; p = 0.0389) at baseline as compared to non-completers.

**Table 1:** Baseline demographic and clinical characteristics of HUB City Steps participants (N=269), Mississippi, 2010

	**n**	**%**
Sex		
Male	40	14.9
Female	229	85.1
Ethnicity		
Hispanic	2	0.7
Non-Hispanic	267	99.3
Race		
African American	254	94.4
White	14	5.2
American Indian/Alaska Native	1	0.4
Marital Status		
Married	113	42.0
Widowed	12	4.5
Divorced	47	17.5
Separated	8	3.0
Never married	89	33.1
Education		
Less than high school	12	4.5
High school graduate/GED	41	15.2
Trade or vocational school	13	4.8
Some college	61	22.7
College degree	76	28.3
Some graduate/professional	19	7.1
Graduate/professional degree	47	17.5
Household income		
<$10,000	40	14.9
$10,000-$19,999	36	13.4
$20,000-$29,999	54	20.1
$30,000-$39,999	37	13.8
$40,000-$49,999	30	11.2
>$50,000	71	26.4
Current smoker	23	8.6
Diagnosed high blood pressure	112	41.6
Diagnosed high blood sugar	42	15.6
Diagnosed high cholesterol	52	19.3
	Mean	SD
Age (years)	44	12.2
Waist circumference (cm)	102	17.6
Body mass index (kg/m^2^)	34.7	8.13
Body fat (%)	42.4	10.20
Fat mass (kg)	42.3	18.79
Lean body mass (kg)	52.4	12.75
Systolic blood pressure (mm Hg)	126	19.2
Diastolic blood pressure (mm Hg)	83	12.3
Total cholesterol (mg/dL)	177	39.1
High density lipoprotein (mg/dL)	52	15.0
Low density lipoprotein (mg/dL)	100	35.4
Triglycerides (mg/dL)	131	79.8
Glucose (mg/dL)	104	37.4
6-minute walk test (distance = m)	440	68.7
SD, standard deviation		

### Pedometer-determined steps/day

Steps/day were reported for over half (55%) of the possible 50,841 participant-days (269 participants x 189 intervention days) in the study. Less than 2% (n=516) of the daily step values were below 500 steps/day, while less than 1% (n=255) were above 30,000 steps/day. On average, participants reported steps/day for 104 (SD = 70 days) of the 189 intervention days, with 41% of participants recording daily step values for at least 75% of the intervention days.

Table 2 contains descriptive statistics for the indicators of pedometer-determined steps/day for all three datasets. For the full dataset, intervention mean and median steps/day were 7,268 and 6,918, respectively. On average, 40% and 26% of the participants’ reported daily steps met at least 7,500 or 10,000 steps/day, respectively. Based on intervention mean steps/day, approximately 25% of the participants fell into each of the limited, low active, and somewhat active step-defined physical activity categories. For change in intervention indicators, the mean and median increases in steps/day were 2,013 and 2,102 steps, respectively, while increases in the six- and four-category step-defined physical activity indicators were 0.7 and 0.5 categories, respectively.

**Table 2:** Descriptive statistics for steps/day indicators of HUB City Steps participants, Mississippi, 2010

	**Mean**	**Median**	**SD**	**Min**	**Max**
*Full dataset (n=27,903 individual steps/day)*					
Mean steps/day	7268	6918	3984	511	30628
Met 7,500 steps/day^a^	40.1	39.0	29.5	0.0	98.5
Met 10,000 steps/day^a^	26.3	16.9	27.2	0.0	97.0
6-category SD-PA (n, %)					
Basal (<2,500 steps)	11	4.6			
Limited (2,500-4,999 steps)	61	25.5			
Low active (5,000-7,499 steps)	69	28.9			
Somewhat active (7,500-9,999 steps)	54	22.6			
Active (10,000-12,499 steps)	29	12.1			
Highly active (≥12,500 steps)	15	6.3			
Change in mean steps/day^b^	2013	1205	3257	−7640	17819
Change in median steps/day^b^	2102	1386	3668	−8656	23730
Change in 6-category SD-PA^b^	0.7	0.0	1.10	−3	4
Change in 4-category SD-PA^b^	0.5	0.0	0.96	−3	3
*Truncated dataset (n=27,903 individual steps/day)*					
Mean steps/day	7221	6914	3721	743	24575
Met 7,500 steps/day^a^	40.1	39.0	29.5	0.0	98.5
Met 10,000 steps/day^a^	26.3	16.9	27.2	0.0	97.0
Change in mean steps/day^b^	1950	1205	3001	−7427	13481
Change in median steps/day^b^	2093	1386	3596	−8464	21422
Change in 6-category SD-PA^b^	0.7	0.0	1.09	−3	4
Change in 4-category SD-PA^b^	0.5	0.0	0.95	−3	3
*Excluded dataset (n=27,132 individual steps/day)*					
Mean steps/day	7279	6972	3417	1276	20637
Met 7,500 steps/day^a^	40.8	40.1	29.5	0.0	98.5
Met 10,000 steps/day^a^	26.6	17.3	27.3	0.0	97.0
Change in mean steps/day^b^	1855	1257	2710	−5931	13237
Change in median steps/day^b^	1954	1344	3084	−5040	15906
Change in 6-category SD-PA^b^	0.7	1.0	1.02	−2	4
Change in 4-category SD-PA^b^	0.6	0.0	0.92	−2	3

SD, standard deviation; Min, minimum; Max, maximum; SD-PA, step-defined physical activity.

^a^ Percentage of values which met at least 7,500 or 10,000 steps/day.

^b^ Change between intervention weeks 1 and 2 and remaining intervention weeks (3–27).

Positive change indicates an increase during intervention.

### Anthropometric and clinical parameter associations

Results for the three pedometer datasets (full, truncated, and excluded) were similar and yielded few meaningful differences in interpretation of the findings. Hence, only results for the full dataset will be reported here. Bivariate associations between steps/day indicators and outcome changes, including anthropometric (waist circumference, BMI, %BF, fat mass, and lean mass), lipid profiles (total cholesterol, HDL, LDL, and triglycerides), glucose, and blood pressure (SBP and DBP) are presented in Table 3. For intervention mean steps/day, a significant negative correlation was observed with %BF (r = −0.15), while a trending (0.05 < P < 0.10) negative correlation was observed with total cholesterol (r = −0.13). Similar results were seen with intervention median steps/day in which correlations with %BF and total cholesterol were both significant (both r = −0.15). For the proportion of reported values meeting at least 7,500 steps/day, significant negative correlations were apparent with changes in total cholesterol and triglycerides, while the negative correlation with change in glucose was trending towards significance (r = −0.16, -0.15, and −0.13, respectively). For the proportion of reported values meeting at least10,000 steps/day, a significant negative correlation was apparent with %BF(r = −0.15); negative correlations with BMI, total cholesterol, and triglycerides were trending towards significance (−0.14 ≤ r ≤ −0.13). No discernible trends were observed among the four- and six-categories of step-defined physical activity indicators and changes in any of the anthropometric or clinical outcome variables (data not shown). For change in intervention mean and median steps/day, significant negative correlations were observed with changes in waist circumference (trending for mean steps/day), BMI, %BF, and fat mass (−0.28 ≤ r ≤ −0.14). Similarly, for changes in intervention four-and six-category classifications, significant negative correlations were observed with changes in BMI, %BF, and fat mass (−0.26 ≤ r ≤ −0.20).

**Table 3:** **Bivariate associations between outcome changes**^**a**^**and steps/day indicators using full dataset: HUB City Steps, Mississippi, 2010**

	**n**	**r**	**P**		**r**	**P**
*Mean steps/day*				*Median steps/day*		
Waist Circumference	178	−0.08	NS	Waist Circumference	−0.10	NS
Body mass index	179	−0.12	NS	Body mass index	−0.12	NS
% Body fat	177	−0.15	0.0469	% Body fat	−0.15	0.0473
Fat mass	177	−0.08	NS	Fat mass	−0.08	NS
Lean body mass	177	−0.05	NS	Lean body mass	−0.05	NS
Total cholesterol	179	−0.13	0.0825	Total cholesterol	−0.15	0.0472
HDL	177	0.01	NS	HDL	0.02	NS
LDL	153	−0.09	NS	LDL	−0.09	NS
Triglycerides	179	−0.12	NS	Triglycerides	−0.11	NS
Glucose	179	−0.11	NS	Glucose	−0.11	NS
SBP	179	0.00	NS	SBP	0.01	NS
DBP	179	0.00	NS	DBP	0.00	NS
*Met 7,500 steps/day*^b^				*Met 10,000 steps/day*^b^		
Waist Circumference	178	−0.06	NS	Waist Circumference	−0.05	NS
Body mass index	179	−0.05	NS	Body mass index	−0.13	0.0940
% Body fat	177	−0.10	NS	% Body fat	−0.15	0.0433
Fat mass	177	−0.03	NS	Fat mass	−0.11	NS
Lean body mass	177	−0.03	NS	Lean body mass	−0.04	NS
Total cholesterol	179	−0.16	0.0317	Total cholesterol	−0.14	0.0638
HDL	177	−0.01	NS	HDL	−0.01	NS
LDL	153	−0.04	NS	LDL	0.00	NS
Triglycerides	179	−0.15	0.0472	Triglycerides	−0.13	0.0741
Glucose	179	−0.13	0.0862	Glucose	−0.09	NS
SBP	179	0.01	NS	SBP	0.03	NS
DBP	179	−0.02	NS	DBP	−0.01	NS
*Change in mean steps/day*^c^				*Change in median steps/day*^c^		
Waist Circumference	165	−0.14	0.0812	Waist Circumference	−0.16	0.0395
Body mass index	166	−0.28	0.0003	Body mass index	−0.27	0.0005
% Body fat	164	−0.26	0.0008	% Body fat	−0.24	0.0020
Fat mass	164	−0.23	0.0027	Fat mass	−0.21	0.0068
Lean body mass	164	−0.05	NS	Lean body mass	−0.06	NS
Total cholesterol	166	−0.10	NS	Total cholesterol	−0.13	NS
HDL	164	0.05	NS	HDL	0.08	NS
LDL	141	−0.10	NS	LDL	−0.12	NS
Triglycerides	166	−0.04	NS	Triglycerides	−0.07	NS
Glucose	166	−0.08	NS	Glucose	−0.07	NS
SBP	166	0.03	NS	SBP	0.03	NS
DBP	166	−0.01	NS	DBP	0.01	NS
*Change in 6-category SD-PA*^*c*^				*Change in 4-category SD-PA*^*c*^		
Waist Circumference	165	−0.10	NS	Waist Circumference	−0.11	NS
Body mass index	166	−0.22	0.0044	Body mass index	−0.20	0.0101
% Body fat	164	−0.26	0.0008	% Body fat	−0.22	0.0052
Fat mass	164	−0.26	0.0006	Fat mass	−0.23	0.0032
Lean body mass	164	0.06	NS	Lean body mass	0.05	NS
Total cholesterol	166	−0.10	NS	Total cholesterol	−0.08	NS
HDL	164	0.05	NS	HDL	0.07	NS
LDL	141	−0.07	NS	LDL	−0.02	NS
Triglycerides	166	−0.03	NS	Triglycerides	−0.06	NS
Glucose	166	−0.06	NS	Glucose	−0.05	NS
SBP	166	0.08	NS	SBP	0.05	NS
DBP	166	0.03	NS	DBP	0.01	NS

r, Pearson's correlation coefficient; SD-PA, step-defined physical activity; HDL, high density.

lipoprotein; LDL, low density lipoprotein; SBP, systolic blood pressure; DBP, diastolic blood pressure.

^a^ Change = baseline subtracted from six-month follow-up.

^b^ Percentage of values which met at least 7,500 or 10,000 steps/day.

^c^ Change between intervention weeks 1 and 2 and remaining intervention weeks (3–27).

Positive change indicates an increase during intervention.

### Multivariate linear regression models

A comparison of the multivariable linear regression analysis results for the three datasets is presented in Table 4. In the presence of significant covariates, various descriptive indicators of steps/day were significant predictors of changes in anthropometric (but not clinical) outcomes. Using %BF as an example, a 1,000 steps/day increase in intervention mean and similar change in intervention mean steps/day resulted in 0.2% decreases in %BF. An increase of 1% in the number of reported days (approximately 2) meeting at least 10,000 steps/day resulted in a 2% decrease in %BF, while a one category change in the six-category step-defined physical activity classification resulted in a 0.6% decrease in %BF. Decreases in anthropometric outcomes were significantly larger for males as compared to females. Age and fitness were significant negative predictors, while BMI and income were significant positive predictors of changes in various anthropometric outcome models. The only significant predictors in the clinical outcome models were fitness (negative for triglycerides and glucose) and diagnosis of relevant condition (positive for glucose). For all of the anthropometric and clinical models, their respective baseline outcome values were significant negative predictors of changes. Marital status, education, smoking status, and compliance with recording steps/day were not significant in any of the models.

**Table 4:** **Multivariable regression results for outcome changes**^**a**^**predicted by steps/day indicators: HUB City Steps, Mississippi, 2010**

		**Dataset**^**b**^						
		**Full**		**Truncated**		**Excluded**		
Outcome	Steps/Day Indicator	β^c^	*P*	β^c^	*P*	β^c^	*P*	Covariates^d,e^
Waist circumference	Change in mean steps/day^f^	−0.33	0.0476	−0.38	0.0419	−0.45	0.0296	BMI, BV
	Change in median steps/day^f^	−0.33	0.0269	−0.33	0.0283	−0.45	0.0123	BMI, BV
Body mass index	Met 10,000 steps/day^g^	−1.05	0.0017	−1.05	0.0017	−1.04	0.0017	Age, BV
	Change in mean steps/day^f^	−0.11	<0.0001	−0.12	<0.0001	−0.13	<0.0001	Age, BV
	Change in median steps/day^f^	−0.10	<0.0001	−0.10	<0.0001	−0.12	<0.0001	Age, BV
	Change in 6-category SD-PA^f^	−0.27	0.0009	−0.28	0.0006	−0.34	0.0001	Age, BV
	Change in 4-category SD-PA^f^	−0.29	0.0020	−0.29	0.0018	−0.34	0.0005	Age, BV
% Body fat	Mean steps/day	−0.15	0.0004	−0.16	0.0006	−0.17	0.0078	Age, sex, BV
	Median steps/day	−0.14	0.0006	−0.14	0.0007	−0.15	0.0084	Age, sex, BV
	Met 10,000 steps/day^g^	−2.23	0.0007	−2.23	0.0007	−2.25	0.0006	Age, sex, BV
	Change in mean steps/day^f^	−0.21	0.0001	−0.22	0.0002	−0.23	0.0004	Age, sex, fitness, BV
	Change in median steps/day^f^	−0.15	0.0016	−0.17	0.0005	−0.20	0.0006	Age, sex, fitness, BV
	Change in 6-category SD-PA^f^	−0.56	0.0005	−0.57	0.0004	−0.64	0.0002	Age, sex, fitness, BV
	Change in 4-category SD-PA^f^	−0.53	0.0036	−0.55	0.0029	−0.70	0.0007	Age, sex, income, fitness, BV
Fat mass	Change in mean steps/day^f^	−0.29	<0.0001	−0.32	<0.0001	−0.35	<0.0001	Age, sex, BV
	Change in median steps/day^f^	−0.25	<0.0001	−0.25	0.0001	−0.30	<0.0001	Age, sex, BV
	Change in 6-category SD-PA^f^	−0.88	<0.0001	−0.90	<0.0001	−1.02	<0.0001	Age, sex, BV
	Change in 4-category SD-PA^f^	−0.90	0.0002	−0.91	0.0001	−1.05	<0.0001	Age, sex, BV
Total cholesterol	Mean steps/day		NS		NS		NS	BV
	Median steps/day		NS		NS		NS	BV
	Met 7,500 steps/day^g^		NS		NS		NS	BV
	Met 10,000 steps/day^g^		NS		NS		NS	BV
Triglycerides	Met 7,500 steps/day^g^		NS		NS		NS	Fitness, BV
	Met 10,000 steps/day^g^		NS		NS		NS	Fitness, BV
Glucose	Met 7,500 steps/day^g^		NS		NS		NS	DX, fitness, BV

BV, baseline value of corresponding outcome variable; SD-PA, step-defined physical activity; NS, not significant at the 0.05 level; DX = diagnosis for relevant condition (high cholesterol, high blood glucose).

^a^ Change = baseline subtracted from six-month follow-up.

^b^ Full included all steps/day values; truncated included all values with those < 500 recorded as 500 steps/day and those > 30,000 recorded as 30,000 steps/day; excluded included only values between 500 and 30,000 steps/day.

^c^ Continuous variable coefficient per 1,000 steps.

^d^ Included age (years), sex, marital status (married or not married), education (less than high school, high school/GED, some college/technical), income (continuous as 12 categories), current smoker, BMI, DX, fitness (baseline value for 6-minute walk test), compliance (proportion of daily step logs completed), and baseline outcome value.

^e^ For all significant outcome models: age, sex (decrease larger for males), fitness, and baseline outcome values had negative associations; income and baseline BMI values had positive associations.

^f^ Change between intervention weeks 1 and 2 and remaining intervention weeks (3–27). Positive change indicates an increase during intervention

^g^ Percentage of values which met at least 7,500 or 10,000 steps/day.

In general, meaningful differences in the magnitude of model effects for the descriptive indicators of steps/day on changes in the anthropometric outcomes were not present when using the full, truncated, or excluded datasets. However, restricting steps/day indicators to include only plausible values may result in stronger predictive ability of intervention mean and median steps/day for changes in waist circumference, and changes in intervention classification of step-defined physical activity for changes in %BF and fat mass. Coefficients of larger magnitude were mostly obtained using the excluded as compared to the full and truncated datasets. For example, a 1,000 steps/day increase in change in intervention mean steps/day resulted in 0.33, 0.38, and 0.45 cm decreases in waist circumference using the full, truncated, and excluded datasets, respectively. Differences in the model coefficient magnitudes ranged from −0.05 to 0.0 between the full and truncated datasets, -0.17 to 0.01 between the full and excluded datasets, and −0.15 to 0.01 between the truncated and excluded datasets.

## Discussion

The primary objective of this secondary analysis of the HUB City Steps data was to evaluate the utility of descriptive indicators of pedometer-determined steps/day for predicting changes in anthropometric and clinical outcomes. Our preliminary findings had established overall significant intervention effects on SBP, DBP, and waist circumference, but not for any other anthropometric or clinical parameter [34]. However, steps/day indicators were not included in these prior analyses. Importantly, this current paper establishes that several steps/day indicators are useful for predicting changes in anthropometric outcomes. For the intervention (computed over the entire intervention period) indicators, mean and median steps/day were significant predictors of change in the %BF models, while the proportion of recorded days meeting at least 10,000 steps/day was a significant predictor of change in the BMI and %BF models. For the change (between first two weeks and remaining intervention weeks) in intervention indicators, changes in mean and median steps/day were significant predictors of changes for all four of the anthropometric outcomes (waist circumference, BMI, %BF, and fat mass), while changes in the four- or six-category step-defined physical activity classifications were significant predictors of changes for BMI, %BF, and fat mass. In all cases, increasing steps/day indicator values were predictive of decreasing anthropometric outcome values. Similar to our results, a trending correlation between increasing change in intervention steps/day and decreasing BMI was observed in a separate six-month community walking intervention conducted in southern African American adults [35]. Likewise, an increase in steps/day was significantly correlated with a decrease in visceral adipose tissue in a walking intervention conducted in obese Japanese men [36]. Further, using categorical increases in steps/day, significant associations were observed with decreases in weight, BMI, hip circumference, total fat mass, %BF, and intra-abdominal fat in a randomized, controlled clinical exercise trial conducted in both men and women [37]. Differing from our results, the significant decrease in BMI reported in a 2007 meta-analysis was not significantly associated with changes in steps/day [25]. The lack of significant associations between changes in steps/day and changes in anthropometric outcomes in the meta-analysis may be partly due to the gross (study) level determination of these associations. That is, direct associations were not computed on an individual participant basis within the studies, but on a gross level as part of the meta-analytic process, effectively reducing the sample size and potentially diluting any effects which may have been present at the individual level.

In contrast to the anthropometric outcomes, none of the steps/day indicators were useful for predicting changes in the clinical outcomes (lipids, blood pressure, and glucose) in the presence of other covariates. Similarly, significant associations between changes in steps/day and changes in LDL and glucose were not found in the meta-analysis [25]. However, while the authors of the meta-analysis did report that intervention participants significantly decreased both their SBP and DBP, the direct association between change in steps/day and change in blood pressure was only trending towards significance (P = 0.08) [25]. In contrast, decreasing triglyceride levels were significantly correlated with increasing steps/day (changes calculated between months one and six) in the separate six-month community walking intervention conducted in southern African American adults [35]. Trending correlations between increasing steps/day and decreasing DBP and increasing HDL were also found [35]. It may be that the lack of association between steps/day indicators and clinical outcome changes in the current study is at least partly due to the participants’ fairly normal baseline clinical values. Further research is warranted to determine if steps/day indicators are useful predictors of intervention-induced changes in related clinical outcomes using more health disparate populations, such as individuals with hypertension, diabetes, or cardiovascular disease.

A secondary objective of this study was to evaluate whether treating steps/day data for implausible values affected the ability of these data to predict intervention-induced changes in clinical and anthropometric outcomes. Since none of the pedometer-determined steps/day indicators were significant in the clinical outcome models, only the results for the anthropometric outcomes will be discussed. We found that treating the steps/day data for implausible values did not appear to affect the magnitude of the model coefficients for predicting intervention-induced anthropometric changes in a meaningful manner. However, with the exception of the BMI model using the proportion of values meeting at least 10,000 steps/day, coefficients of greater magnitude were obtained using the excluded compared to the full and truncated datasets. The largest differences in model coefficients among the three datasets were observed for the intervention mean and median steps/day predicting changes in waist circumference, and changes in intervention classification of step-defined physical activity categories predicting changes in %BF and fat mass. It is not clear why these differences were found and no supporting data could be identified in the literature. It is possible that direct measures of body fat, including central adiposity, are more sensitive to implausible steps/day values than are other anthropometric measures such as BMI. Excluding implausible values reduces the variability present in the extremities of steps/day distributions which may strengthen relationships between steps/day indicators and some anthropometric outcome changes. Exploratory analyses using pedometer data from a subset of participants in this study who reported at least one implausible steps/day value suggest this may be true as differences between model coefficients using full and excluded datasets were magnified for waist circumference, %BF, and fat mass (but not for BMI) in this subset compared to the full set of participants (data not shown).

### Study limitations and strengths

The main limitations of the present study are the self-report nature of the steps/day data and the lack of information on the intensity or speed of participant walking. Despite accumulating steps, it is feasible that the intensity and/or duration of walking bouts were not sufficient to achieve health benefits or changes in anthropometric or clinical outcomes. Evidence suggests that additional health benefits are possible from participation in higher intensity and/or longer duration physical activity [38]. As previously mentioned, there was no true baseline steps/day data collected as part of the original study design, a limitation that most directly impacts this secondary analysis. Nevertheless, due to the continuous recording implemented during the intervention, we were able to describe change from the first two weeks through the remaining intervention weeks. The magnitude of change apparent from this analysis is similar to increases reported in three meta-analyses conducted on pedometer-based intervention studies which did include baseline assessments [16,25,39]. Further, it is possible that some of the increase in steps/day observed in this study may have been partly due to seasonal changes [40] since data collection began in the winter and ended in the summer. The non-fasted status for the blood lipids and glucose measures is also a limitation, particularly for triglycerides [41]. Finally, we recognize that anthropometric and clinical outcome changes could have been attributed to intervention components other than physical activity, such as dietary changes and motivational interviewing. However, exploration of these components in conjunction with the extensive analysis of the pedometer-determined steps/day is beyond the scope of this paper and should be addressed in future research. Despite these limitations, the strengths of this secondary analysis are derived from the original study design that included the community-based (i.e. “real life”) nature of the study, the option of either paper or web-based recording of pedometer logs (as preferred by the participant), and the collection of steps/day for the entire length of the intervention (vs. the more typical limited number of days at the beginning and end of the study).

## Conclusions

These results suggest that several descriptive indicators of steps/day may be useful for predicting changes in anthropometric outcomes in a southern US, African American, adult population. While both intervention (computed based on data collected over the entire intervention period) and change (from first two weeks to remainder of intervention weeks) in intervention steps/day indicators were significant predictors of changes in anthropometric measures, only the change in intervention steps/day indicators were useful for all four anthropometric outcomes (waist circumference, BMI, %BF, and fat mass). In general, meaningful differences in the magnitude of model effects for the steps/day indicators on changes in the anthropometric outcomes were not present when implausible steps/day were either truncated or excluded compared to the model coefficients obtained with the full set of values. These results suggest that manipulating steps/day data to address implausible values has little overall effect on the ability to predict intervention-induced anthropometric changes. Further research is warranted as these results indicate that the approach used in the handling and analysis of pedometer-determined steps/day data may depend upon the study outcomes measured. Until then, we suggest that change in mean steps/day may be the most useful descriptive indicator for predicting changes in anthropometric outcomes due to its association with a number of anthropometric measures as well as its intuitively acceptable interpretation.

## Abbreviations

6MWT: Six minute walk test; %BF: Percent body fat; BMI: Body mass index; DBP: Diastolic blood pressure; HDL: High density lipoprotein; LDL: Low density lipoprotein; SBP: Systolic blood pressure; US: United States.

## Competing interests

The authors declare that they have no competing interests.

## Authors’ contributions

JT performed the data analysis, interpreted the results, and drafted the manuscript. AS was head of the data collection team, helped with data transfer and interpretation, and contributed to manuscript revision. JZ conceptualized and designed the original study, conceptualized the objectives of the manuscript, worked out the Methods section, and contributed to data interpretation. CT-L wrote parts of the Discussion and Conclusions, and contributed to data interpretation and manuscript revision. MW contributed to manuscript revision. CC and KY conceptualized and designed the original study and contributed to manuscript revision. All authors read and approved the final manuscript.

## Pre-publication history

The pre-publication history for this paper can be accessed here:

http://www.biomedcentral.com/1471-2458/12/983/prepub
